# *GmFULc* Is Induced by Short Days in Soybean and May Accelerate Flowering in Transgenic *Arabidopsis thaliana*

**DOI:** 10.3390/ijms221910333

**Published:** 2021-09-25

**Authors:** Jingzhe Sun, Mengyuan Wang, Chuanlin Zhao, Tianmeng Liu, Zhengya Liu, Yuhuan Fan, Yongguo Xue, Wenbin Li, Xiaoming Zhang, Lin Zhao

**Affiliations:** Key Laboratory of Soybean Biology of Ministry of Education China, Northeast Agricultural University, Harbin 150030, China; jingzhesun0306@126.com (J.S.); wmy8977@126.com (M.W.); z1390421338@126.com (C.Z.); ltm0729@126.com (T.L.); xiaochengai2021@126.com (Z.L.); fyh1404207035@126.com (Y.F.); xyg81@126.com (Y.X.); wenbinli@neau.edu.cn (W.L.)

**Keywords:** soybean, *GmFULc*, photoperiod, flowering time, *TPL*

## Abstract

Flowering is an important developmental process from vegetative to reproductive growth in plant; thus, it is necessary to analyze the genes involved in the regulation of flowering time. The MADS-box transcription factor family exists widely in plants and plays an important role in the regulation of flowering time. However, the molecular mechanism of *GmFULc* involved in the regulation of plant flowering is not very clear. In this study, GmFULc protein had a typical MADS domain and it was a member of MADS-box transcription factor family. The expression analysis revealed that *GmFULc* was induced by short days (SD) and regulated by the circadian clock. Compared to wild type (WT), overexpression of *GmFULc* in transgenic *Arabidopsis* caused significantly earlier flowering time, while *ful* mutants flowered later, and overexpression of *GmFULc* rescued the late-flowering phenotype of *ful* mutants. ChIP-seq of GmFULc binding sites identified potential direct targets, including *TOPLESS* (*TPL*), and it inhibited the transcriptional activity of *TPL*. In addition, the transcription levels of *FLOWERING LOCUS T* (*FT*), *SUPPRESSOR OF OVEREXPRESSION OF CONSTANS1* (*SOC1*) and *LEAFY* (*LFY*) in the downstream of *TPL* were increased in *GmFULc-* *overexpression* *Arabidopsis*, suggesting that the early flowering phenotype was associated with up-regulation of these genes. Our results suggested that GmFULc inhibited the transcriptional activity of *TPL* and induced expression of *FT*, *SOC1* and *LFY* to promote flowering.

## 1. Introduction

Soybean [*Glycine max* (L.) Merrill] is a typically SD plant, as its vegetative and reproductive growth are closely related to the photoperiod [1]. The soybean switches from vegetative growth to reproductive growth only after the length of daylight is shortened to a critical limit. Due to the photoperiod sensitivity characteristic of soybean, when it is planted at higher latitudes, where it is exposed to long-day (LD) conditions in the growing season, they often show late flowering, the growth period is prolonged, and they might not even flower or mature normally; conversely, when it is planted at lower latitudes, where it is exposed to shortened lengths of daylight in the growing season, it generally shows early flowering, shortening of the growing period, reduction of yield and even failure of normal growth. The flowering and maturity of soybean are seriously affected by the length of the growing season in the planting region, so that the geographical adaptative region of soybean varieties are generally narrow, and the photoperiod response characteristic is an important factor affecting the regional adaptability of soybean varieties [2,3,4]. Therefore, it is necessary to clarify the molecular mechanism of some genes involved in the photoperiod pathway, in order to provide a basis for the cultivation of soybean varieties that have a relatively wide regional adaptability.

Many members of the MADS-box transcription factor family, which play key roles in plant development processes, including flowering induction, flowering time, formation of floral meristems and organs, fruit formation and seed pigmentation, such as *FUL*, *APETALA1* (*AP1*), *CAULIFLOWER* (*CAL*), *SEPALLATA* (*SEP*) *1/2/3* and *AGAMOUS* (*AG*), are involved in the formation of flowers, and MADS-box proteins can form heterodimers with other proteins to regulate flowering [5,6,7]. The floral organ identity gene *FUL* in *Arabidopsis* contains a MADS-box domain, also known as *AGL8*(*AGAMOUS-LIKE8*), which belongs to the *AP1/FUL*-like gene subfamily with *AP1* and *CAL* [8,9]. The *FUL* gene has broader functions compared to *AP1* and *CAL*: it promotes the formation of inflorescence, dividing tissue in early flower development, but also in the late flower development pericardial and young horn fruit, and also regulates leaf development [10]. In the photoperiod-dependent flowering pathway, *CONSTANS* (*CO*) integrates various photoperiod regulatory genes, and the activated *FT* is expressed in the leaf vascular bundles. *FT* is then transported to the apical meristems, triggering the expression of *FUL* and *SOC1* in the apical meristems. *FUL* is involved in the regulation of the transformation from flower bud meristems to inflorescence meristems, and then *FUL* and *SOC1* are expressed in the developing inflorescence meristems layer to induce the transformation to floral meristems, thus controlling the flowering time [11]. Expression of florigen gene *FT* is induced by LD in the leaves, and then the FT is transported from leaves to apical meristems and binds to the FD protein. The FT/FD protein complex in the apical meristems promote the expression of the characteristic gene *FUL/SOC1* of the floral meristems to induce flowering [12,13]. *FUL* induces flowering by antagonizing the effect of *FLC* flowering time inhibitor, SVP interacts with FLC protein to inhibit flowering and FUL interacts with SVP protein to antagonize the inhibitory effect of FLC on flowering, after which FUL interacts with SOC1 protein to form FUL and SOC1 dimers that bind to the *LFY* promoter, thereby promoting *LFY* expression and inducing flowering [14,15]. The conserved sequence of MADS-box protein and DNA binding is generally CC (A/T) _6_GG, called CArG-box [16].

Similar to *AtFUL*, rice *FUL*-type factors are involved in flower initiation as characteristic genes of floral meristems. *PAP2* is a member of the SEPALLATA subfamily, and it interacts with MADS14 and MADS15 to promote the transition of vegetative meristems to inflorescence meristems. Different from *Arabidopsis*, rice *FUL*-type factors also act in the leaves upstream of *Hd3a/RFT1* and participate in the regulation of flowering time [17]. During tomato ripening, *FUL1* and *FUL2* act on the upstream of the ethylene signal pathway to promote fruit ripening [18,19,20]. In cucumber, *CsFUL1^A^* is a gain of function allele in long-fruited cucumber and its expression level manipulating fruit length [21]. Soybean MADS-box gene, *AGAMOUS-LIKE1*(*GmAGL1*) played a role in floral organ formation and dehiscence, and promotes flowering in the photoperiod pathway [22,23]. *GmFLC-like (FLOWERING LOCUS C)* is the member of FLC clade of the MADS-box transcription factor family and participates in late flowering triggered by long-term low temperature by inhibiting the expression of *FT* [24]. Recent studies have found that in the photoperiod pathway, GmGBP1 interacting with GmGAMYB might regulate flowering time by up-regulating the transcriptional level of *GmFULc* [25,26]. *TPL* participates in various molecular pathways, such as the circadian clock, flowering time regulation, auxin, jasmonic acid and ethylene hormone signaling in *Arabidopsis* [27,28,29]. The *tpl* mutant decreased photoperiod sensitivity and showed early flowering by reducing the expression level of *FT* [30]. The aim of our study is to clarify the molecular mechanism of *GmFULc*, promoting flowering through the soybean photoperiod effect, further providing a new way for improving the growth period of soybean.

In this study, *GmFULc* was identified as a member of the MADS-box transcription factor family. Transgenic *Arabidopsis* with *GmFULc* overexpression has the early flowering phenotype. ChIP-seq predicted the possible target gene *TPL* and preliminarily confirmed that GmFULc promotes flowering by inhibiting the activity of *TPL*. In addition, *GmFULc-ox* increased the expression of flowering-related genes *FT*, *SOC1* and *LFY*.

## 2. Results

### 2.1. Sequence Analysis of the GmFULc

**Figure 1:**
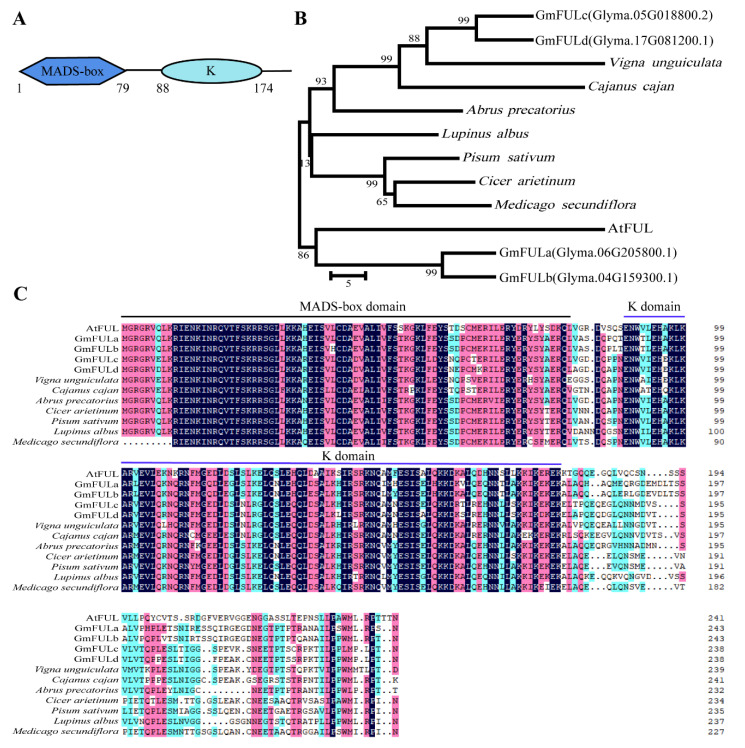
Sequence and phylogenetic tree analysis and subcellular localization of GmFULc. (**A**) Predicted domains of GmFULc. (**B**) Phylogenetic relationships of GmFULc and some FUL proteins from other species. Accession numbers are listed in Supplementary Table S1. Phylogenetic tree containing four soybean proteins with the complete MADS-box domain and eight FUL proteins from *Arabidopsis thaliana*, *Vigna unguiculata*, *Cajanus cajan*, *Abrus precatorius*, *Cicer arietinum*, *Pisum sativum*, *Lupinus albus* and *Medicago secundiflora*. Phylogenetic tree was constructed using the neighbor joining method with 1000 bootstrap replicates by MEGA 6.0. (**C**) Sequence alignment of GmFULc and other FUL proteins from other species. The MADS and K-domain were indicated by the line on the top. The black line and the blue line mark the MADS-box domain (1–79 aa) and the K-domain (88–174 aa), respectively. Light blue, homology ≥ 50%; pink, homology ≥ 75%; blue, homology 100%.

*GmFULc* (*Glyma.05G018800.2*) might play a crucial role in promoting flowering in soybean [25]. The gene was cloned from soybean variety “DongNong 42” according to the referencing Phytozome database (https://phytozome.jgi.doe.gov/pz/portal.html, accessed on 1 April 2021). The cDNA sequence of *GmFULc* is 1149 bp, containing the ORF of 720 bp, encoding 239 amino acids, and the predicted molecular weight was 27.55 kDa. GmFULc has a MADS domain at the N-terminal, followed by a K-domain (keratin-like), which is predicted at amino acid residues 1–79 and 88–174, respectively (Figure 1A). Phylogenetic tree containing four soybean proteins and eight FUL proteins from other species was constructed. GmFULc/d were classified into the one branch with *Vigna unguiculata* (XP_027922086.1), *Cajanus cajan* (XP_020227938.1), *Abrus precatorius* (XP_027333786.1), *Cicer arietinum* (XP_004508656.1), *Pisum sativum* (AFI08227.1), *Lupinus albus* (KAE9601902.1) and *Medicago secundiflora* (AFU81360.1) (Figure 1B). These indicate that the protein encoded by GmFULc/d may be a relatively conserved protein in leguminous crops. AtFUL is closely related to GmFULa/b, indicating that GmFULa/b may have similar biological functions to AtFUL (Figure 1B). The evolution distance between GmFULc/d and GmFULa/b was relatively far, indicating that there may be some differences in the function of FUL proteins in soybean. We compared the amino acid sequence of GmFULc with the FUL homologous proteins of other species. The sequence of MADS domain of GmFULc is highly conserved among some species, while the conservation of K-domain was much weaker (Figure 1C).

### 2.2. Photoperiod and the Circadian Clock Regulate the Expression of GmFULc

**Figure 2:**
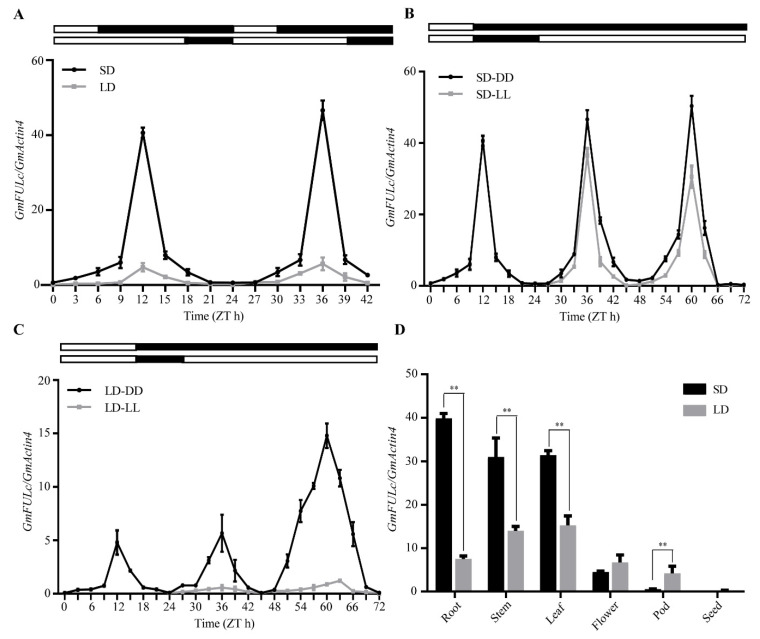
*GmFULc* expression patterns analysis. A photoperiod-sensitive soybean variety, “Dongnong 42”, was grown in LDs (16 h light/8 h dark) for 10 days and then transferred to SDs (8 h dark/16 h light) or LDs for sampling at 3-h interval. (**A**) The expression levels of *GmFULc* in SDs and LDs. (**B**) Expression patterns of *GmFULc* transcripts under LL and DD conditions from SDs. (**C**) Expression patterns of *GmFULc* in LL and DD conditions from LDs. White and black bars at the top represent the light and dark phases, respectively. (**D**) Tissue-specific expression of *GmFULc* at ZT 12 h in SDs and LDs. Data are means ± standard errors of the mean (SEM) of three independent experiments. Significant differences between the expression level of *GmFULc* under SDs and LDs are indicated by asterisks. Student’s *t*-test, ** *p* *<* 0.01.

Soybean trifoliate leaves grown for 20 days were collected every 3 h and the daily expression pattern of *GmFULc* under SDs and LDs was analyzed by qRT-PCR. *GmFULc* showed photoperiod specific expression patterns in SDs and LDs. The expression levels of *GmFULc* in SDs were higher than LDs (Figure 2A). The expression pattern of *GmFULc* was analyzed after SDs and LDs transfer under constant light (LL) and darkness (DD) conditions. Under the condition of SDs-DD and SDs-LL, the level of *GmFULc* mRNA maintained a strong rhythm and reached the peak at ZT 12 h (Figure 2B,C). Therefore, *GmFULc* was regulated by the circadian rhythm and induced by SDs in soybean leaves.

### 2.3. The tissue-specific Expression Patterns of GmFULc in Soybean

In order to study the tissue-specific expression patterns of *GmFULc* during growth and development of soybean, different tissues of soybean (root, stem, leaf, flower, pod and seed), treated with SDs and LDs, were collected 12 h after dawn. The expression level of *GmFULc* was the highest in the roots and leaves under SDs and LDs, respectively. In roots, stems and leaves, the expression level of *GmFULc* was higher in SDs than that in LDs. There was no significant difference of *GmFULc* mRNA abundance in seeds between SDs and LDs (Figure 2D).

### 2.4. Overexpression of GmFULc May Accelerate Flowering in Transgenic Arabidopsis

**Figure 3:**
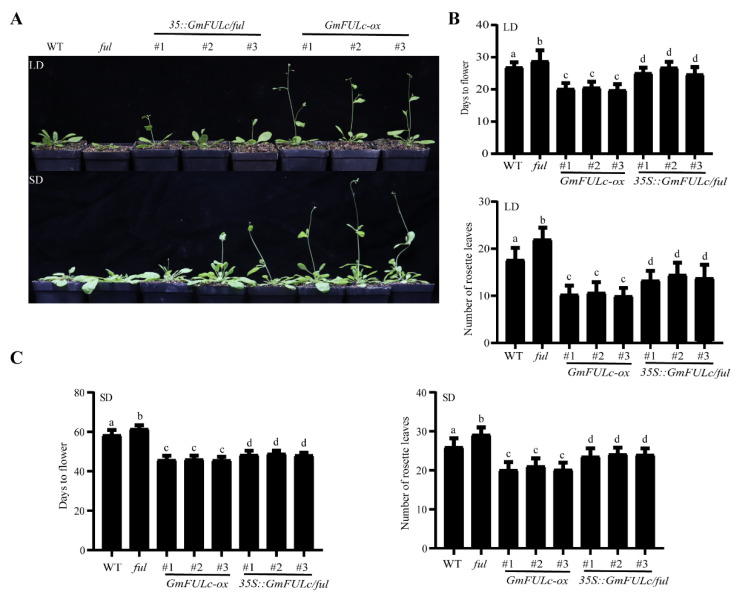
Phenotypes of transgenic *Arabidopsis* in SDs and LDs. (**A**) Flowering phenotype of *GmFULc-ox*, *ful* mutant, *35S:GmFULc*/*ful* restoration and WT plants in SDs and LDs. Photographed after the plants were grown in SDs and LDs for 55 days and 28 days, respectively. (**B**) Days to flower and number of rosette leaves of *GmFULc-ox*, *ful* mutant, *35S:GmFULc/ful* and WT in LDs. (**C**) Days to flowering and number of rosette leaves of *GmFULc-ox*, *ful* mutant, *35S:GmFULc/ful* and WT in SDs. Data are shown as means ± SEM. For each experiment, three technical replicates were conducted. One-way ANOVA was used to generate the *p* values. The same letter denotes nonsignificant differences across the two panels (*p* > 0.05).

*GmFULc* was transformed into *Arabidopsis* under the control of a CaMV 35S promoter and *GmFULc-ox* transgenic *Arabidopsis* were obtained. The flowering phenotypes of WT, *GmFULc-ox, GmFULc-ox*/*ful* and *ful* mutants were observed in SDs and LDs. The flowering time of *GmFULc-ox* transgenic *Arabidopsis* were advanced by 6 days in LDs, while flowering time of *ful* mutants was delayed by 2 days. Complementary experiments on *ful* mutants were carried out. The expression of *35S:GmFULc* rescued the late-flowering phenotype of *ful* mutants. The number of rosette leaves of *GmFULc-ox* transgenic *Arabidopsis* and *ful* mutants were less and more than that of WT during bolting, respectively (Figure 3A,B). The flowering time of *GmFULc-ox* was earlier than that of *ful* mutants by 5 days in SDs (Figure 3A,C). The results showed that overexpression of *GmFULc* promote flowering in transgenic *Arabidopsis*.

### 2.5. Chromatin Immunoprecipitation Sequencing Assays of GmFULc-Target Genes

**Figure 4:**
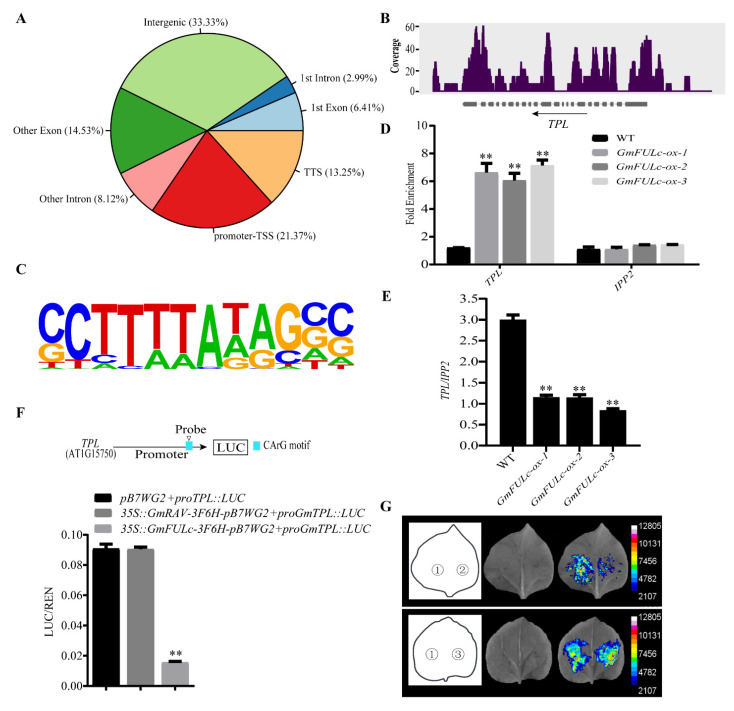
Validation and expression analyses of the selected GmFULc target genes. (**A**) The binding sites are distributed relative to the position of the target genes. (**B**) Peak diagram showing the designated gene loci in the ChIP-seq raw reading in the integrated genome observer. The arrow indicates the direction of the transcription. (**C**) Motif analysis of *GmFULc*-binding sequences using Hypergeometric Optimization of Motif EnRichment (HOMER) software. (**D**) ChIP-qPCR from *GmFULc-ox* transgenic *Arabidopsis* and WT plants. (**E**) The mRNA abundance of *TPL* in the 14-day-old plants. (**F**) Upper panel: the physical location of the base sequence fragments. The effect of GmFULc on the *TPL* promoter activity. Relative LUC activity of cotransfected reporter and effector in *N. benthamiana* leaves. Data are shown as means ± SEM of three independent experiments. Student’s *t*-test, ^∗∗^ *p* *<* 0.01. (**G**) The LUC activities of *TPL*. 1: *pB7WG2* + *proTPL:LUC* were used as the blank control; 2: *35S:GmFULc-3F6H-pB7WG2* + *proTPL:LUC*; 3: *35S:GmRAV-3F6H-pB7WG2* + *proTPL:LUC* was used as the negative control; D-luciferin was used as the substrate of LUC.

The target genes of GmFULc were identified by ChIP-seq technique to further clarify the possible mechanism of *GmFULc* promoting flowering. Among the 234 GmFULc binding sites detected, 182 (77.77%) sites were located at gene regions. Among the 182 sites in genic regions, 21.37%, 20.94%, 11.11% and 13.25% were located in defined promoter regions, exon, intron and TTS, respectively (Figure 4A). *TPL* (*AT1G15750*) might be a candidate target gene for GmFULc. In *Arabidopsis*, *TPL* plays a role in inhibiting flower transition in the upstream of floral integrator *FT* [30].

The GmFULc binding site of the *TPL* genome is located at the promoter (Figure 4B). According to the common sequences of GmFULc binding sites detected, the possible GmFULc binding motifs were predicted by HOMER software [31]. According to the predicted results, we determined that GmFULc binds to the target gene *TPL* motif CArG-box (*p*-value = 1×10^−4^) (Figure 4C). AtFUL regulated *SAUR10* by binding conserved CArG-box of its promoter [32]. The promoter of *TPL* contained the CArG-box, which was similar to the CArG-box combined with the AtFUL and *SAUR10* promoter. In the ChIP-qPCR experiment, the fragments containing GmFULc binding sites in the *TPL* promoter region were highly enriched in ChIP with Anti-FLAG, indicating that GmFULc binds to the *TPL* promoter (Figure 4D).

### 2.6. GmFULc Inhibits the Transcriptional Levels of TPL

ChIP-qPCR found that the *TPL* promoter was bound by GmFULc, and qRT-PCR found that the mRNA abundance of *TPL* was decreased in *GmFULc-ox Arabidopsis*. Reporter *proTPL:LUC* was constituted of a *TPL* promoter driving the *LUC* reporter gene. The LUC activity of co-infiltrating *pB7WG2* and *proTPL:LUC* was lower than co-infiltrating *35S:GmFULc-3F6H-pB7WG2* and *proTPL:LUC* into *N. benthamiana* leaves, indicating that GmFULc inhibit the expression level of *TPL*. The LUC activity with the floral repressor GmRAV [33] and *proTPL:LUC* co-transformed the leaves of *N. benthamiana*, which showed no difference compared with the control (Figure 4F,G). In summary, GmFULc significantly inhibited the transcriptional levels of *TPL* by directly binding to its promoter.

### 2.7. GmFULc Affects the Transcriptional Levels of Flowering Time Related Genes

**Figure 5:**
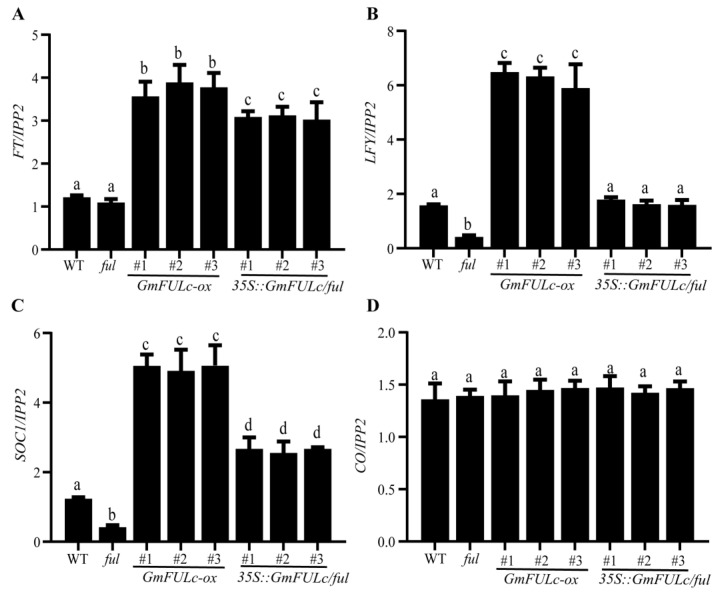
The transcriptional levels of *FT*, *LFY, SOC1* and *CO* in *GmFULc-ox*, *ful* mutants, *35S:GmFULc/ful* and WT plants. (**A**–**D**) *GmFULc-ox*, *ful* mutants, *35S:GmFULc/ful* and WT plants were planted in LDs, and sampled to determine the transcriptional levels of *FT*, *LFY, SOC1* and *CO* at ZT 12 h. Data are shown as means ± SEM of three independent experiments. For each experiment, three technical replicates were conducted. One-way ANOVA was used to generate the *p* values. The same letter denotes nonsignificant differences across the two panels (*p* > 0.05).

Overexpression of *GmFULc* led to early flowering in *Arabidopsis*. In order to further investigated the regulation mechanism of *GmFULc* on flowering time, the transcriptional levels about flowering-related genes (including *FT*, *LFY, SOC1* and *CO*) in 14-day-old plants. The expression levels of *FT*, floral meristems recognition *LFY* and floral integration *SOC1* in transgenic plants with *GmFULc-ox* were significantly higher than that in WT plants, but *LFY* and *SOC1* significantly decreased in *ful* mutants. In *35S:GmFUL/ful*, the down-regulated trend of *SOC1* and *LFY* were rescued (Figure 5A–C). The transcriptional levels of *CO* were not significantly different from that of WT (Figure 5D). *FT*, *LFY* and *SOC1* play roles in the downstream of TPL [30]. In summary, GmFULc inhibited the expression of *TPL*, and induced the transcriptional levels of *FT, LFY* and *SOC1* to promote flowering in *Arabidopsis*.

## 3. Discussion

Members of the MADS-box transcription factor family contain a highly conserved domain called the MADS-box domain, which are widely found in plants [34]. Some members of the MADS-box family have been reported in *Arabidopsis*, rice, tomato and cucumber [15]. However, the function of *GmFULc* has not been reported in soybean. In this study, *GmFULc* was isolated and identified from soybean. GmFULc was identified as a member of the MADS-box family. In addition, the MADS-box domain between the GmFULc and FUL proteins from some species shared high conservation (Figure 2B,C). The AP1/FUL subfamily of MADS-box genes may originate from repeated events before the differentiation of existing angiosperms [35].

The transcript levels of *GmFULc* were relatively more expressed in stems, but at lower level in seeds of soybean (Figure 2D). *FUL* is negatively regulated by AP1 in the early stages of *Arabidopsis* development. *FUL* is almost not expressed in the early vegetative organs; until *Arabidopsis* transits to reproductive growth, *FUL* is strongly expressed in the inflorescence meristems, and its expression patterns are different from soybean [8]. Although GmFULc and AtFUL have amino acid homology, there may be some evident differences in biological function that need to be further clarified. Analysis of *GmFULc* expression pattern suggested that *GmFULc* was regulated by the circadian rhythm and induced by SDs in soybean leaves.

The homologue gene of *GmFULc*, *GmFULa* expression level was affected by photoperiod conditions, but not affected by diurnal rhythm [36].

Overexpression of *GmFULc* in *Arabidopsis* showed early flowering and the *ful* mutants showed late flowering in SDs and LDs. Compared with the *ful* mutant, the phenotype of the *35S:GmFULc/ful* complementary line were rescued (Figure 3A). Therefore, the transcriptional level of *GmFULc* was regulated by day length and might participate in the regulation of flowering at *Arabidopsis*. The loss of *AtFUL* function led to delayed flowering in SDs and LDs, while the overexpression of *AtFUL* caused an early flowering phenotype [10,11]. Furthermore, the overexpression of *GmFULc* in *Arabidopsis* led to a significantly early flowering phenotype compared with the wild type (Figure 3A). The transcriptional levels of *LFY, SOC1* and *FT* increased significantly in transgenic *Arabidopsis* (Figure 5).

In the photoperiod pathway, *FUL* is target of FT and FD function during flowering. The high expression level of FT further increases the expression of *FUL* and leads to morphological changes in leaves. The accumulation of FUL in meristems is related to the transition of flowering [37]. Different from *Arabidopsis*, rice is an SD plant, and thus, *SEPALLATA* MADS-box gene *PAP2* and the *AP1/FUL*-like genes (*MADS14*, *MADS15* and *MADS18*) were located upstream and induced expression of *Hd3a* and *RFT1* in rice leaves. *AP1/FUL*-like genes play different roles to promote reproductive transition. The FT is transported from leaves to SAM, while *PAP2*, *MADS14*, *MADS15* and *MADS18* were induced in SAM. These regulations might help to accelerate the changing meristems phase by magnifying the florigen signal [17]. The distinct ways that *AtFUL* and *GmFULc* function could be due to differences between species. The working mode of *GmFULc* is similar to that of the *AP1/FUL*-like genes in rice. These may be the reason that transcript levels of *GmFULc* were higher than that of *AtFUL* in the leaves, but this speculation has not been confirmed. The mechanism of *GmFULc* regulating soybean flowering might be different from that of *AtFUL*, which needs to be clarified. This suggests that both *GmFULc* and *AtFUL* promote the flowering of *Arabidopsis*, and they may be functionally distinct in some aspects [15].

The expression of *GmFULa* and *GmFULb* (soybean FUL homolog genes) were inhibited by *GmFT1a* and delayed flowering in soybean [38]. It has been suggested that different genes in the same family may perform different functions in soybean, and this needs to be clarified. In order to confirm the potential targets of GmFULc, *GmFULc-ox* transgenic *Arabidopsis* were used to predict the target genes of GmFULc by ChIP-seq. By screening the genes in which GmFULc binds to the promoter region, a flowering-related gene, *TPL*, was identified. In *Arabidopsis*, TPL was a transcription mediator in various molecular pathways, such as flowering time regulation, leaf development, anthocyanin accumulation, auxin and jasmonate signaling transduction pathway [27,28,29,39,40,41,42]. The formation of the CDF–TPL complex reduces the expression of *FT, LFY* and *SOC1* play roles in the downstream of TPL [30]. A CArG-box *CTTTTAAGG* is located at the *TPL* promoter region, similar to the reported *AtFUL* binding motif *CCAAATATGG* in the *SAUR10* promoter region [32]. GmFULc reduced the transcriptional activity of *TPL* and *GmFULc-ox* transgenic *Arabidopsis* showed the expression of *TPL* decreased significantly when the expression of *LFY, SOC1* and *FT* increased. *GmFULc* can promote flowering by repressing *TPL* transcriptional activity in *Arabidopsis.*

## 4. Materials and Methods

### 4.1. Plant Materials, Growth Conditions and Records of Data

For the analysis of GmFULc, we used seeds from a photoperiod-sensitive soybean variety, “DongNong 42”. Seeds were planted in growth chambers at 28 °C in LDs (16 h light/8 h dark), and when expanding the trifoliate leaves, partial plants were transferred to SDs (8 h light/16 h dark) under the same environmental conditions with LDs. The seedlings were sampled at every 3-h interval under SDs and LDs (transferred in 15 days), continuous light (LL) and dark (DD). Samples were collected under SDs and LDs from different tissues, including root, stem, leaf, flower, pod and seed. *Arabidopsis thaliana* (Columbia-0) was used for genetic transformation. Seeds from the ABRC were obtained from the *ful* mutant (SALK_033647). *GmFULc-ox*, *ful* mutant, *35S:GmFULc/ ful* and WT seeds were planted on 1/2MS agar medium. Seeds were vernalized for 3 days and transferred to the growth chambers (22 °C), and 12-day-old seedlings were transferred to 1:1 of vermiculite and turfy-soil for flowering phenotype analysis at SDs and LDs. *GmFULc-ox*, *35S:GmFULc/ ful* and WT were cultured under the LDs of 1/2MS agar medium, and qRT-PCR analyses were carried out for flowering-related genes. Flowering time was measured by the number of days from germination to bolting. Means ± SEM deviation was used in the statistical analysis of the data. At least 20 plants were analyzed for each cultivar and each time, and the experiments were repeated three times.

### 4.2. Plasmid Construction and Generation of Transgenic Arabidopsis Plants

The *pENTRY-3F6H* included the 3×FLAG and 6×His [43]. The PCR products were cloned into *pENTRY-3F6H* linearized by *Sma*I using In Fusion cloning to construct recombinant *35S:GmFULc-3F6H-pENTRY* and recombinant vector *35S:GmFULc-3F6H -pB7WG2* was transformed into WT and *ful* mutants by the floral dip method [44] using the *Agrobacterium GV3101*. Seeds were selected with 6 mg/L phosphinothricin on 1/2 MS agar medium. Three homozygous lines of T_3_ transgenic seeds were chosen for further research.

### 4.3. ChIP-Seq and ChIP-qPCR

*GmFULc-ox-1* transgenic *Arabidopsis* plants were mixed and sampled, and then 0.6 g seedlings were frozen. ChIP experiments were performed as previously described [45]. IP were performed using Anti-FLAG antibody (Monoclonal ANTI-FLAG®M2 antibody, F1804, Sigma-Aldrich, Saint Louis, MO, USA) and Anti-IgG antibody (Normal Rabbit IgG antibody, 2729, Cell Signaling Technology, Danvers, MA, USA). The DNA was sent to a sequencing company for sequencing.

### 4.4. Transient Assay of TPL Promoters Affected by GmFULc in N. Benthamiana

To generate *TPL* promoter-driven *LUC* constructs *proTPL:LUC*, the promoter of *TPL* was amplified from genomic DNA of *Arabidopsis* using *proTPL:LUC*-F and *proTPL:LUC*-R primers (Table S2). The PCR product was purified and cloned into binary vector *pGreenII-0800-LUC*, linearized by *Sma*I using In-Fusion cloning system (Takara, Beijing, China). The constructs *35S:GmFULc-3F6H-pB7WG2* and *proTPL:LUC* were simultaneously transferred by *Agroinfection* into *N. benthamiana*. A floral repressor *GmRAV* effector construct (*35S:GmRAV-3F6H-pB7WG2*) and *proTPL:LUC* were transferred into *N. benthamiana* as the negative control [43].

### 4.5. Quantitative RT-PCR Analysis

RNA isolation was performed as previously described [46]. QRT-PCR amplifications were performed using the TransStart^®^ Tip Green qPCR SuperMix (TransGen Biotech, Beijing, China) on applied Biosystems^TM^ 7500 Fast Dx Real-Time PCR Instrument (Applied Biosystems, Foster City, CA, USA). The PCR cycling conditions: 94 °C for 30 s; 40 cycles of 94 °C for 5 s; 50 °C for 34 s. *IPP2* (AT3G02780) and *GmActin4* (GenBank accession number AF049106, National Center for Biotechnology Information, USA) were used as reference genes of *Arabidopsis* and soybean, respectively. The primers used in qRT-PCR analyses are shown in Table S2. Three biological replicates and three technical replicates were applied for all experiments.

### 4.6. Statistical Analysis

At least three biological replicates were included in the data, and all data were analyzed using Student’s *t*-test or ANOVA, for the determination of the significant differences with *SPSS* 25.0 (IBM, Armonk, NY, USA). All data were analyzed using *GraphPad* Prism 8.0.2 (*GraphPad* Software, San Diego, CA, USA) for calculating mean and standard errors of the mean.

## 5. Conclusions

In conclusion, GmFULc binding with the promoter of target gene *TPL* and repressed the transcriptional of *TPL*, then weakened the inhibitory effect of TPL on *FT* and promoting the flowering. FT interacts with FD to activate downstream floral organ genes to induce flowering [47]. It is necessary to further investigate the molecular regulatory mechanism of *GmFULc* in soybean.

## Data Availability

The ChIP-seq data names of the repositories and accession numbers can be found below: NCBI-SRA database under the BioProject no. PRJNA743583 and accession Nos. SRR15047998 and SRR15047997.

## Supplementary Materials

The following are available online at https://www.mdpi.com/article/10.3390/ijms221910333/s1.
