# Supplementary material for: GmFULc Is Induced by Short Days in Soybean and May Accelerate Flowering in Transgenic Arabidopsis thaliana
Source: Int J Mol Sci. 2021 Sep 25;22(19):10333. doi: 10.3390/ijms221910333 (PMC8508813; doi:10.3390/ijms221910333)
Supplement: Supplementary file 1 [file ijms-22-10333-s001.zip › Supplementary table1 and 2.pdf]

Table S1. Plant FUL proteins' information.

Species name	Accession number
Arabidopsis thaliana	AT5G60910.1
Glycine max	Glyma.06G205800.1
Glycine max	Glyma.04G159300.1
Glycine max	Glyma.05G018800.2
Glycine max	Glyma.17G081200.1
Vigna unguiculata	XP_027922086.1
Cajanus cajan	XP_020227938.1
Abrus precatorius	XP_027333786.1
Cicer arietinum	XP_004508656.1
Pisum sativum	AFI08227.1
Lupinus albus	KAE9601902.1
Medicago secundiflora	AFU81360.1

Table S2. The specific sequences of the primers.

Primer	Sequence (5'-3')
GmFULc-3F6H-F	TGGAGCTCGGTACCCATGGGGAGGGGAAGAGTG
GmFULc-3F6H-R	TCAGCAGGGATCCCCAATTCATTTGTAGGAAGAGGCATC
GmRAV-3F6H-F	TGGAGCTCGGTACCCATGGATGGAGGCTGTGTCACAG
GmRAV-3F6H-R	GATCCTGGGATCCCCGACAAAGCTCCAATTACTTTTAACTTC
qAtTPL- ChIP-F	TCTGTTAATGGAATCTAGCTGTTTC
qAtTPL- ChIP-R	TTGTGAACAGTCTCTTTAAACTTCT
proTPL::LUC-F	GAATTCCTGCAGCCCTTTTAACAAAGTACAGTTTGCTTTC
proTPL::LUC-R	ACTAGTGGATCCCCCGTTTTCTCTCACTTCCTTAAAAGAC

qAtTPL -F	GAGTTTTATGGATGCAACAGTTTG
qAtTPL-R	TAGTGGATGTACGTTTGAATTGCT
qGmActin4 -F	GTGTCAGCCATACTGTCCCCATTT
qGmActin4-R	GTTTCAAGCTCTTGCTCGTAATCA
qGmFULc-F	GAGCAAGAAGGACTGCAAAATA
qGmFULc-R	TTGTAGGAAGAGGCATCAAAGG
qAtIPP2-F	TGAGCTTGGTATTGTAGCTGAA
qAtIPP2-R	GACAGTTTCAAACCTTCCTCAC
qAtCO-F	AAGGTGATAAGGATGCCAAGGAG
qAtCO-R	GGAGCCATATTTGATATTGAACTGA
qAtFT-F	TGGTGGAGAAGACCTCAGGAAC
qAtFT-R	TGCCAAGCTGTCGAAACAATAT
qAtSOC1-F	TCAGAACTTGGGCTACTC
qAtSOC1-R	TTCTCGTCGTCTCCGCCTCC
qAtLFY-F	TGTGAACATCGCTTGTCGTC
qAtLFY-R	TAATACCGCCAACTAAAGCC
